# Spontaneous Coronary Artery Dissection: A Literature Review

**DOI:** 10.7759/cureus.45868

**Published:** 2023-09-24

**Authors:** Angelo A Messina Alvarez, Mohammad A Bilal, Ahmad R Damlakhy, Nouraldeen Manasrah, Ahmed Chaudhary

**Affiliations:** 1 Internal Medicine, Detroit Medical Center/Sinai-Grace Hospital, Detroit, USA

**Keywords:** management, diagnosis, types, incidence, scad

## Abstract

Spontaneous coronary artery dissection is a medical condition characterized by the rupture of the coronary artery wall, occurring without any external trauma. This ailment has been linked to various inflammatory, rheumatologic, and connective tissue disorders, as well as pregnancy-related changes. Despite being a less familiar cause of acute coronary syndrome, it has a considerable mortality rate, with incidence rates reaching up to 4%. This review will discuss the occurrence, pathophysiology, categorization, risk factors, diagnostic techniques, and treatment approaches related to spontaneous coronary artery dissection.

## Introduction and background

Spontaneous coronary artery dissection (SCAD) was initially reported in 1931 by Pretty [1]. The diagnosis was made in a woman in her early 40s who displayed symptoms of acute coronary syndrome upon admission to the hospital [1]. SCAD is characterized by the sudden non-iatrogenic rupture of the coronary artery wall, resulting in the formation of a false lumen within the arterial wall layers. This rupture can occur due to internal or external forces. Regardless of the underlying cause, it impairs blood flow to the coronary artery, ultimately leading to myocardial ischemia [2].

The clinical presentation of SCAD shares multiple overlapping features with acute myocardial infarction (MI), as both conditions fall under the category of acute coronary syndrome (ACS). While diagnostic approaches and management options are often similar in both cases, it should be noted that they cannot be regarded as completely interchangeable. Certain therapeutic interventions used for MI may result in harm to SCAD patients [3].

SCAD was previously believed to be an extremely rare condition. However, it is now being increasingly recognized as a potential cause of acute coronary syndrome, especially in young female patients. Physicians are advised to consider SCAD as a possible differential diagnosis when a young patient presents to the emergency room with typical chest pain and lacks classic risk factors for coronary artery disease, ultimately leading to an acute ischemic event. Despite this increased recognition, there is still a paucity of literature and data available on the acute and long-term management of this condition [4]. The objective of this study is to enhance awareness and provide further insights into the current literature regarding the incidence of SCAD, diagnostic options, and treatment opportunities to improve the outcomes of patients who present to the hospital with this often unrecognized diagnosis.

## Review

Anatomic and histologic overview

The coronary system comprises two primary vessels: the left main coronary artery (LMCA) and the right coronary artery (RCA), originating from the Valsalva sinuses within the initial segment of the ascending aorta. The LMCA can measure up to 25 mm in length before bifurcating into the left anterior descending (LAD) artery, which can extend up to 13 mm, and the left circumflex (LCX) artery, which can extend up to 8 mm. Conversely, the RCA typically has a length of 14 mm before transitioning into the posterior descending artery (PDA) [5]. The trajectory of these arteries is significant as it determines the areas they supply with blood, reflected in the electrocardiogram (EKG) during ischemic events. A table outlining the areas of the heart supplied by each artery is provided below (Table 1).

**Table 1:** Basic coronary anatomy and perfusion areas

Branch of Coronary Artery	Perfused Territory
Left anterior descending (LAD) artery	Base of interventricular septum (anterior half) and anterior part of the left ventricular free wall [5]
Left circumflex artery	Lateral wall of the left ventricular wall [5]
Right coronary artery	Anterolateral, posterior area of right ventricle [5]
Posterior descending artery	Apex of the interventricular septum (posterior half) and posterior part of left ventricular free wall [5]

The histological structure of the coronary artery wall is similar to that of other vascular structures and comprises four layers: the innermost layer known as the "luminal" layer, the "tunica intima" layer, the "tunica media" layer, and the outermost layer known as the "tunica adventitia." The tunica intima, the second layer, is composed of endothelial cells, connective tissue, and smooth muscle cells, providing a smooth lining to the vessel and serving as a barrier. Additionally, this layer has a metabolic/endocrine function that produces antithrombotic, prothrombotic, fibrinolytic, and anti-inflammatory agents. The third layer, tunica media, consists of smooth muscle cells and connective tissue containing collagen fibers and nerve axons that allow for neural stimulation and neurotransmitter diffusion, leading to vasoconstriction or vasodilation. The outermost layer, tunica adventitia, is composed of fibrous tissue and is surrounded by vasa vasorum, nerves, and lymphatic vessels. This outer layer plays a crucial role in the development of SCAD [5].

Pathophysiology

SCAD is characterized by the formation of an intramural hematoma within the coronary artery walls. Two proposed mechanisms for the development of SCAD are the "inside-out theory" and the "outside-in theory." The first theory involves a rupture in the subendothelial layer that leads to the formation of a false lumen, which is then aggravated by coronary blood flow and results in an expanding intramural hematoma. The second theory involves a spontaneous rupture of the vasa vasorum in the adventitia or tunica media, resulting in the formation of a hematoma that also expands over time [6]. Regardless of whether the intramural hematoma develops through the "inside-out" or "outside-in" theory, both mechanisms result in obstruction of coronary blood flow due to the expansion of the hematoma, ultimately leading to myocardial infarction because of supply-demand mismatch [7].

Classification

Spontaneous coronary artery dissection has been classified into three different groups based on angiographic findings (Table 2).

**Table 2:** Spontaneous coronary artery dissection classification

Type	Description
Type 1	Arterial wall is stained by contrast, and there is evidence of multiple radiolucent lumens [8].
Type 2	Long, diffuse stenosis for more than 20 mm. Type 2a: There is a normal lumen before and after the hematoma extension [8]. Type 2b: The hematoma extension prolongs until the distal part of the artery with no presence normal lumen after [8].
Type 3	Focal stenosis that extends for less than 20 mm [8].

The vessel most frequently affected by SCAD is the left anterior descending artery which can be disturbed in 60% of the cases. However, there is a significant prevalence of SCAD in the left circumflex artery in up to 38% of the cases, while less often right coronary artery and left main coronary artery have represented a case of SCAD (29% and 12%, respectively) [8].

Incidence

In the past, SCAD was known to have a rare occurrence in ACS, estimated at only 0.2%, while it has been identified in 0.5% of sudden cardiac death occurrences. This cardiac ailment mainly affects women (80%) with a median age between 45 and 56, coinciding with menopausal years. The highest prevalence of SCAD occurs in the Caucasian race, which accounts for 82% of reported cases [9]. SCAD is still not diagnosed frequently enough, particularly in young and healthy women. However, the utilization of high-sensitivity troponin, the accessibility of invasive angiography, and the awareness of medical professionals have led to an increase in the identification of SCAD cases [9].

Risk factors

SCAD is a multifactorial condition that arises due to a combination of several triggering factors. These factors include the presence of arteriopathies, which, in combination with significant emotional or physical stress, can result in circulatory shear force that leads to SCAD. Activities such as Valsalva maneuvers, coughing, vomiting, or straining, which increase thoracoabdominal pressures, or the intake of sympathomimetics like cocaine, can cause catecholamine surges due to intense physical activity, also leading to SCAD [10].

The second set of factors that can trigger SCAD are hormonal influences, such as prolonged exposure to exogenous estrogen and progesterone, or imbalances in thyroid function, which can alter the vascular wall and eventually lead to SCAD [8]. Additionally, proinflammatory states or chronic inflammatory conditions, such as systemic lupus erythematosus (SLE), rheumatoid arthritis (RA), inflammatory bowel disease (IBD), sarcoidosis, and other vasculitis characterized by the infiltration of inflammatory cytokines and cells into the vessel wall, can impair the resistance of blood flow within the vessel and contribute to the development of SCAD [10].

Connective tissue disorders, such as Marfan syndrome and Ehlers-Danlos syndrome, can disrupt the collagen fibers present in the coronary layers, making individuals more susceptible to SCAD [10]. Nevertheless, the most significant risk factor for SCAD in the general population is fibromuscular dysplasia (FMD), a non-atherosclerotic, non-inflammatory vasculopathy characterized by abnormalities in the composition of the arterial wall. Recent studies between 2011 and 2022 indicate that FMD is present in up to 86% of patients with SCAD [10].

Spontaneous coronary artery dissection and pregnancy

SCAD is the most frequent cause of ACS during pregnancy, accounting for an estimated prevalence of 40% in ACS cases. It is believed that significant hemodynamic and hormonal changes during pregnancy make individuals more susceptible to SCAD, and this predisposition can persist until the puerperium period. Furthermore, each pregnancy represents an additional risk factor, with the cumulative effect increasing the long-term risk of SCAD, making it a latent risk factor for coronary dissection [11].

A population-based study involving over 4 million pregnancies revealed an incidence of SCAD in 1.8 per 100,000 pregnancies. Of the total cases, 85% initially presented to the hospital with features of ACS and were diagnosed through coronary angiography [12]. A literature review that included 1,547 cases of SCAD in women revealed that 33% of cases (510 cases) occurred in pregnant women. Although SCAD presents with less severe cardiac symptoms, it poses a significant risk to both the mother and the baby [13].

Clinical presentation

The manifestation of SCAD varies depending on the extent of the hematoma, the rate of development, and the degree of injury. The most frequent symptom is typical cardiac chest pain that is retrosternal in location, has a pressure quality, and can radiate to the jaw and left arm. The pain is typically exacerbated by exertion and may be accompanied by diaphoresis, anxiety, palpitations, and changes in blood pressure [14]. Patients may develop myocardial infarction or cardiogenic shock when there is significant obstruction. Meanwhile, rupture and leakage of the vessel can result in cardiac tamponade. Sudden cardiac death (SCD) is observed in approximately 50% of patients, and these cases have been linked to dissection of the left main coronary artery [15].

Diagnosis

The initial assessment of SCAD typically relies on electrocardiography (EKG) as patients commonly present with symptoms suggestive of ACS. In a meta-analysis conducted by Giacoppo et al., ST-elevation myocardial infarction was observed in 48% of cases, while non-ST-elevation myocardial infarction was found in 36.3% of patients, unstable angina in 6.5%, and 5.5% of patients presented with stable angina or ventricular arrhythmias [16].

Invasive angiography is the preferred method for diagnosing SCAD, regardless of how it initially presents. A classic sign of SCAD on imaging is the presence of multiple lumens within a vessel and extraluminal contrast. SCAD can be further classified into three groups based on angiographic findings: Type 1, which is the least common, is characterized by numerous lumens within the vessel and is seen in 20% of cases. Type 3 resembles tubular narrowing from atherosclerosis. Type 2 is the most common and is characterized by a single lumen that can be bounded by normal vessel anatomy (2a) or extend to the distal area of the artery (2b), seen in up to 57% of cases. During angiography, vessel tortuosity is also evaluated as it is predictive of recurrent SCAD [17].

Intravascular ultrasound is another approach for diagnosing SCAD and is preferred when invasive angiography is not diagnostic or contraindicated. Currently, it is used to differentiate between type 3 SCAD and atheromatous plaque and to assist with coronary intervention by aiding in the selection of stent size and placement. However, it is important to note that any invasive imaging method carries the risk of exacerbating SCAD or causing iatrogenic coronary artery dissection [17].

Finally, computed tomography coronary angiography is a non-invasive method that allows for visualization of the arterial wall and lumen. However, it is not considered the first-line diagnostic approach for SCAD for two main reasons. Firstly, it has a lower resolution than invasive angiography, decreasing sensitivity. Secondly, it does not allow in situ intervention compared to invasive methods [17].

Management

Conservative approach

Conservative management has been shown to be effective in follow-up angiography after 35 days from the major event. Observational data has demonstrated that up to 97% of patients who undergo conservative management experience healing from the original SCAD lesion. In a prospective study conducted in Vancouver, 232 patients with SCAD were managed conservatively, and only 3.5% of the cases required revascularization, specifically 2.9% underwent PCI, and 0.6% underwent coronary artery bypass grafting (CABG) [18].

The mainstay of conservative therapy for SCAD is antiplatelet therapy and beta-blockers. Beta-blockers reduce shear stress on the vessels, which is why they are also used in aortic dissection. Antiplatelet therapy is essential in preventing thrombus formation in the place of intramural hematoma and is also necessary for patients with ACS who will require revascularization, as they need to have dual antiplatelet therapy. Aspirin is considered the cornerstone of primary and lifelong secondary prevention, while a second antiplatelet agent, such as P2Y12 inhibitors, is recommended for 12 months regardless of whether the patient underwent PCI or not [18].

The role of anticoagulation in SCAD is uncertain. Although it is theorized to prevent thrombus formation, its use carries a significant risk of increased bleeding and progression of coronary dissection. Therefore, anticoagulation should be discontinued as soon as the diagnosis of SCAD is confirmed via angiography. Thrombolytic therapy should also be avoided in cases of high suspicion for SCAD. In a study of 87 patients with SCAD who received fibrinolysis before the diagnosis was confirmed, there was a significant worsening of the condition that required emergent rescue interventions [19].

Finally, the role of statins in the management of SCAD is uncertain since patients generally do not have significant atherosclerosis. However, they are recommended in patients with SCAD who have concomitant atherosclerosis or hyperlipidemia. The use of angiotensin-converting enzyme inhibitors (ACEIs)/angiotensin II receptor blockers (ARBs) is considered when there is impaired heart systolic function after the coronary event (ejection fraction (EF) < 50%) [19].

Invasive approach

The decision to perform invasive intervention in SCAD patients is based on various factors such as clinical symptoms, angiographic results, and the experience of the medical team. The classification and location of the SCAD lesion are also important in determining the need for intervention and the type of procedure to be performed. Patients with severe coronary occlusion and associated myocardial ischemia, cardiogenic shock, or ventricular arrhythmias should be considered for revascularization [20].

PCI in patients with SCAD poses several difficulties due to the fragile state of the coronary artery and risks such as dissection, hematoma extension, and wire entry into the false lumen. In cases where PCI is not feasible, coronary artery bypass grafting (CABG) may be considered, especially in situations such as left main coronary artery dissection, two-vessel dissection, or PCI failure. It is important to note that the main objective of revascularization in SCAD patients is not to repair the coronary anatomy but rather to ensure adequate coronary blood flow [20]. Nevertheless, revascularization is related to poor procedural success rates and high rates of complications. Overall, coronary artery revascularization should be avoided, especially in patients with low-risk features or no major indications for intervention [20].

Prognosis and mortality

Female patients generally have a poorer prognosis than male patients with SCAD, especially pregnant women, who are at a higher risk. Specifically, SCAD that develops during the puerperium period has been associated with the worst prognosis. Furthermore, recent analysis has revealed that the in-hospital mortality rate for SCAD can be as high as 5%, and after one year, the mortality rate remains similar at around 4%. However, when analyzing long-term mortality over 10 years, the mortality rate increases to 7.7% [21].

It is worth emphasizing that as technology has advanced and angiography has become more widely available; mortality rates for ACS secondary to SCAD have improved. For example, Vogiatzis and colleagues reported that between 1993 and 2003, there was a 28% decrease in mortality rates for this condition, with rates dropping from 66% to an unspecified lower rate during that time period [22].

## Conclusions

SCAD is an infrequent yet increasingly recognized etiology of ACS, primarily due to heightened awareness and greater access to diagnostic modalities. Patients typically exhibit clinical features reminiscent of acute myocardial infarction (AMI), including retrosternal chest pain that radiates to the left arm and jaw, accompanied by sweating, shortness of breath, and apprehension. SCAD is associated with a substantial mortality rate, but meanwhile, its incidence is on the rise, and the mortality rates have been declining.

The diagnostic algorithm for SCAD follows the ACS protocol, with EKG serving as the primary diagnostic tool, and angiography as the definitive diagnostic modality for confirming the type of SCAD, guiding subsequent management. Conservative management is generally preferred, as it has demonstrated a favorable prognosis compared to invasive interventions, which are associated with worse outcomes.

Nevertheless, invasive intervention remains the optimal therapy for complications such as hemodynamic instability, cardiogenic shock, or vessel-specific injury. The future of SCAD management and research should concentrate on determining optimal invasive strategies that enhance procedural outcomes and ascertaining the utility of pharmacologic therapies typically employed in AMI management but may not confer advantages in SCAD.
